# Mycetoma: a clinical dilemma in resource limited settings

**DOI:** 10.1186/s12941-018-0287-4

**Published:** 2018-08-10

**Authors:** Pembi Emmanuel, Shyam Prakash Dumre, Stephen John, Juntra Karbwang, Kenji Hirayama

**Affiliations:** 1 Department of Immunogenetics, Institute of Tropical Medicine (NEKKEN), Nagasaki University, Nagasaki, Japan; 2 Program for Nurturing Global Leaders in Tropical and Emerging Communicable Diseases, Graduate School of Biomedical Sciences, Nagasaki University, Nagasaki, Japan; 3 Hospital Services Management Board Yola, Adamawa State Ministry of Health, Yola, Nigeria; 4 Adamawa State Agency for HIV/AIDS Control, Yola, Nigeria; 5 Department of Clinical Product Development, Institute of Tropical Medicine (NEKKEN), Nagasaki University, 1-12-4 Sakamoto, Nagasaki, 852-8523 Japan

**Keywords:** Mycetoma, Eumycetoma, Actinomycetoma, Clinical dilemma, Dermatological diseases, Social stigma, Disability, Neglected tropical diseases

## Abstract

**Background:**

Mycetoma is a chronic mutilating disease of the skin and the underlying tissues caused by fungi or bacteria. Although recently included in the list of neglected tropical diseases by the World Health Organization, strategic control and preventive measures are yet to be outlined. Thus, it continues to pose huge public health threat in many tropical and sub-tropical countries. If not detected and managed early, it results into gruesome deformity of the limbs. Its low report and lack of familiarity may predispose patients to misdiagnosis and delayed treatment initiation. More so in situation where diagnostic tools are limited or unavailable, little or no option is left but to clinically diagnose these patients. Therefore, an overview of clinical course of mycetoma, a suggested diagnostic algorithm and proposed use of materials that cover the exposed susceptible parts of the body during labour may assist in the prevention and improvement of its management. Furthermore, early reporting which should be encouraged through formal and informal education and sensitization is strongly suggested.

**Main text:**

An overview of the clinical presentation of mycetoma in the early and late phases, clues to distinguish eumycetoma from actinomycetoma in the field and the laboratory, differential diagnosis and a suggested diagnostic algorithm that may be useful in making diagnosis amidst the differential diagnosis of mycetoma is given. Additionally, a proposed preventive measures which may be helpful in the community is also provided. Since treatment is currently based on expert opinion, we encourage active research to establish treatment guideline for it.

**Conclusion:**

Since delay in visiting health facility results into gruesome complication, early presentation, recognition and initiation of appropriate choice of regimen is helpful in reducing complications. The clinical overview of mycetoma and the suggested algorithm may enhance suspicion and possibly increase recognition of mycetoma in the community and further guide in differentiation of eumycetoma from actinomycetoma. There is an urgent need for research funding for mycetoma, a disease plagued by severe physical disabilities and social stigma leading to isolation.

**Electronic supplementary material:**

The online version of this article (10.1186/s12941-018-0287-4) contains supplementary material, which is available to authorized users.

## Background

Mycetoma continues to pose huge public health threat in many tropical and sub-tropical countries [1, 2]. If not detected and managed early, it causes gruesome deformity of the limbs, severe disability, premature termination of occupation, difficulty in finding jobs and partner among young adults and accelerates the rate of drop outs from school in children [1]. Just like leprosy, in which stigma affects many dimensions of victim’s life [3], mycetoma is also plagued by various socio-economic consequences [4]. Though uncertainty hovers on its actual global incidence, a field survey from West Nile State of Sudan, a country uniquely believed to be the most endemic in the world, yielded a prevalence of 14.5/1000 population in 2010 [1, 5]. Several autochthonous and imported cases have also been reported from Europe [6–8]. Prolonged diagnostic delays complicate mycetoma as seen in a patient (infected with *Aspergillus nidulans*) from Senegal with a diagnostic delay of 15 years [9]. Despite its several worrisome impacts, mere mentioning of mycetoma still steers an astonishment. This is probably due to insufficient awareness of its existence as a consequence of low recognition and reporting [4] as it is not a reportable disease [10]. This situation may predispose patients to misdiagnosis [11] and eventual mistreatment [12] in a scenario where limitation of diagnostic tools prevails, and health personnels are left handicapped with little or no option but to clinically diagnose the patients [13]. Mycetoma still faces additional challenges such as lack of standardized treatment and strategic public health control guidelines by the World Health Organization (WHO) making its management to be exclusively based on physician’s discretion.

Since the clinical outcome of mycetoma is associated with disease severity [14], it is truism that early commencement of appropriate regimen will reduce disability or disfigurement [1]. This can be achieved through prompt and accurate identification of mycetoma amidst other diseases mimicking its presentation [15] and careful distinction of eumycetoma from actinomycetoma [13]. In this review we seek to give an overview on the clinical presentation of mycetoma in early and late phases which may assist in recognising the disease in its various forms of appearance, to distinguish actinomycetoma from eumycetoma and the two from other differentials so that appropriate management can be initiated or referral to higher facility undertaken timely. Additionally, a suggested algorithm to augment knowledge in making diagnosis in the resource-limited fields is proposed. Diagnostic tools are also highlighted to help guide investigation to make accurate diagnosis in facilities which possess such tools. A suggested control measure which can be used in educating the community thereby reducing their exposure to risk factors during field work has been equally offered.

## Distribution of mycetoma

### Geographical areas

Mycetoma usually occurs within 15° south to 30° north of the equator [1, 2, 16–20] situated around the tropic of cancer [15, 21–24]. This area includes India, Yemen, Saudi Arabia, Mexico, Venezuela, Argentina, Colombia and Brazil [2, 25–31]. In Africa, cases are seen in Sudan, Nigeria, Mauritania, Ethiopia, Chad, Kenya, Djibouti, Cameroon, Somalia, Tunisia, Niger and Senegal [32–38]. Mycetoma is also reported from Laos, Singapore, Malaysia, Philippines, Indonesia, Cambodia, Thailand and Vietnam [17]. Occasional reports of mycetoma appears in temperate regions of European countries such as Germany, Albania, Bulgaria, Greece, Italy, and Turkey [11, 39–46]. In France, mycetoma has been reported in migrants from Africa [7, 8]. Sporadic cases have also been reported from subtropical regions of south-western United States [47]. In general, mycetoma is believed to have much wider distribution but this is hampered by misdiagnosis and isolation of cases as seen in a case report from south Africa [48].

Eumycetomas predominantly occur in Africa and Southern Asia with *Madurella mycetomatis* being the most frequent eumycete accounting for 70% of all cases in Sudan [22]. On the other hand, Actinomycetomas are predominately seen in the Americas with *Nocardia brasiliensis* being the most prevalent organism responsible for 86% of all cases in Mexico [21–23]. A retrospective study in Mexico reported a cumulative of 3933 cases of mycetoma, of which 97% were actinomycetoma while only 3.5% were eumycetoma [1].

### Aetiology and host of mecetoma

Currently, over 56 taxonomically varying organisms, either of fungal (eumycetoma) or bacterial (actinomycetoma) origin have been implicated as causative agents of mycetoma [1] (Additional file 1). Most frequent organisms causing actinomycetoma are *Streptomyces somaliensis*, *Actinomadura madurae*, *A. pelletieri*, *Nocardia brasiliensis* and *N. asteroides*, while the most common pathogens reported in eumycetoma are *M*. *mycetomatis*, M. *grisea*, *Pseudoallescheria boydii and Leptosphaeria senegalensis* [49].

Mycetoma occurs in all age groups and is most often seen in the age range of 20–40 years [1, 2, 50] but rarely seen in children. More males than females are affected [15] and it commonly occurs in field labourers and cultivators whose occupation involves direct contact with the soil [16, 50]. No known vector or animal reservoir has been established, however mycetoma in animals has been described to be either infected experimentally or naturally [1]. In humans, mycetoma naturally progresses from early to chronic phase.

## Pathogenesis

Though several people are exposed to the causative agents of mycetoma, only few come down with the disease [23]. An inter play of pathogen, host and environment occurs in the pathogenesis of mycetoma [49]. Inoculation of the etiologic agents (which usually thrive well in the favorable climatic conditions of the mycetoma belt) occurs when human makes contact with soil, thorn, etc. during manual activities [23, 49]. Initially a local host response characterized by a nonspecific inflammatory response and neutrophil chemotaxis occur [23]. This non-specific response subsequently becomes more organized and cellular [49]. Macrophages and monocytes migrate to the site of infection and their micro-biocidal activities are enhanced by cytokines stimulation (interferon gamma and tumor necrosis factor alpha) [23]. A study has demonstrated that following stimulation of peripheral blood mononuclear cells by *M. mycetomatis antigen*s, T helper (Th)2-like responses [interleukin (IL)-10 and IL-4] are produced in primary lesions, and in draining lymph nodes in *S. somaliensis* infection [49]. Mahgoub et al. studied the T cell-mediated immune response to eumycetoma fungi with a subsequent claim that eumycetoma patients presented a weak cell-mediated response [51]. Protective effect of antibodies has been shown to be produced by IgM but not IgG in experimental mice [49]. The role of genetics has also been studied and found chitotriosidase, an enzyme, to be responsible for the pathogen-eliminating immune response by binding to fungal chitin in mycetoma grain. Thus its polymorphism which results in decreased activity of chitotriosidase enzyme was found to be associated with increased likelihood of developing eumycetoma [52].

## Clinical presentation

**Fig. 1:**
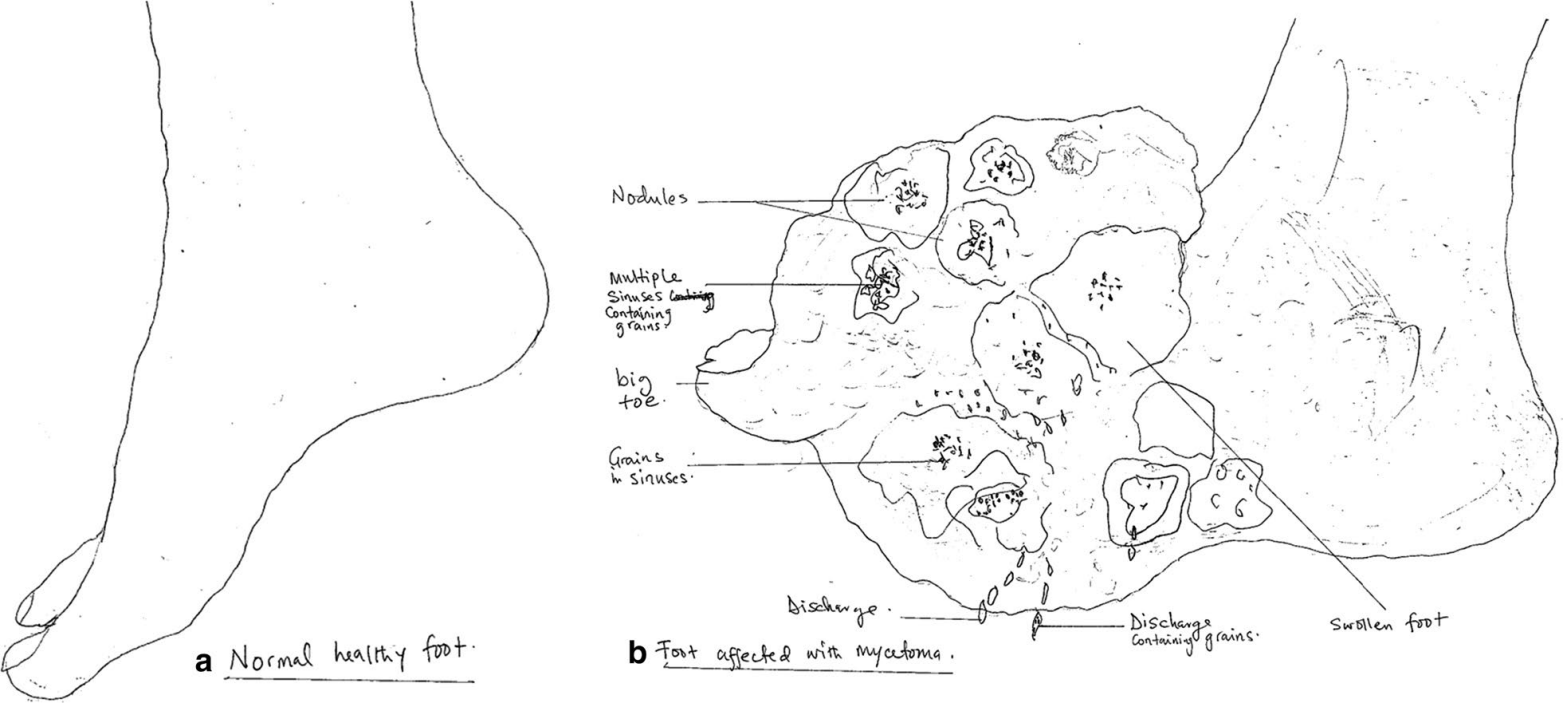
Schematic diagram showing: **a** a normal foot and **b** foot disfigured by the destructive nature of mycetoma due to delay in instituting clinical management. Figures are schematic presentation and may not scale to actual measurements

Characteristic features of eumycetoma and actinomycetoma closely resemble each other and their differentiation is required for proper management. Their characteristics features are shown in Table 1 while a concise clinical differentiation between eumycetoma and actinomycetoma is provided in Table 2. Figure 1 shows a normal foot and the foot disfigured by the destructive nature of mycetoma due to delay in instituting clinical management.

**Table 1:** Characteristic features of eumycetoma and actinomycetoma

Characteristics	Eumycetoma	Actinomycetoma
Causative agents	Fungi [32]	Bacteria [32]
Geography	Common in Africa and India [1]	Common in Latin America [1]
Occupation	Field workers [1]	Field workers [1]
Age group	Common in 20–40 years [1]	Common in 20–50 years [1]
Anatomical parts affected	Usually hand, feet and other parts of arms and legs [1]	Usually chest, abdomen and head [1]
Course of progression	Slow [64]	More rapid and inflammatory [64]
Sinus (number, morphology)	Few, proliferative, protuberant [1]	Many, depressed, flat [1]
Fistula	Few [32]	Many [32]
Bone invasion	Delayed [32]	Rapid [32]
Bone cavities on radiograph	Fewer but larger with clear margins [32]	Numerous, small with unclear margins [32]
Lymphatic spread	Occassional [1]	Frequent [1]
Veins proximal to lesion	Commonly dilated	Seldom dilated
Grains size	Larger (0.5–2 mm) [64]	Smaller (20–100 μm) [64]
Grain texture	Coarse [64]	Fine [64]
Pigment	Melanin [64]	Absent [64]
Hyphae [64]	Septate (4–5 μm thick)	Fine, branching filaments (< 1 μm)
Acid fast staining	Negative [64]	Weakly acid fast (e.g. *Nocardia*)
Masson–Fontana silver staining	Positive [64]	Negative [64]
PAS staining	Positive [64]	Negative [64]
GMS staining	Positive [64]	Positive [64]
B and B staining	Negative [64]	Gram positive [64]
Ultrasound features	Hyperechogenic [1]	Less echogenic [1]
Treatment	Drugs (antifungal) + surgery [1]	Drugs (antibiotics) [1]

*GMS* Grocotts Methenamine silver Stain, *B and B* Brown and Brenn stain, *PAS* periodic-acid Schiff staining

**Table 2:** Usefulness and pitfalls of diagnostic tools used in mycetoma

Diagnostic methods	Usefulness	Pitfalls
Clinical	Utilised in endemic areas where diagnostic facilities are lacking It boosts referral	Does not identify the etiologic agent Does not reveal the spread of disease along the different tissue planes and bone
Imaging		
X-ray	Can determine the extent of lesions Multiple features can be detected Help plan treatment strategy Can be used in low-resource settings once expertise is available	Requires expert for interpretation
Ultrasound	Determine the extent of lesions Differentiate between mycetoma and non-mycetoma lesions Differentiate between eumycetoma and actinomycetoma Help plan appropriate treatment strategy Can be used in low-resource settings once expertise is available	Cannot differentiate between different causative agents Not readily available in the field or peripheral hospitals Requires expert for interpretation
MRI	Determine the extent of lesions Fast and non-invasive Help plan treatment strategy	Unsuitable for discrimination Available only in tertiary facilities Requires highly expert persons
CT	Determine the extent of lesions Discriminate eumycetoma between actinomycetoma It is fast and non-invasive Help plan appropriate treatment strategy	Not specific for early bony involvement Available only in tertiary facilities Requires highly expert persons
Laboratory		
Microscopy	Cheaper and easy to use Can be utilised in the field and resource constraint settings Gram stain can distinguish fungal from bacteria agents Lacto-phenol cotton blue stain can differentiate fungal from bacterial filaments Acid fast stain helps identify the positive hyphae of *Nocardia*	Cannot identify specific etiologic agents
Culture	Gold standard for aetiology identification Aids in proper management of patients	Time consuming, contamination is common, high expertise needed Mostly available only in tertiary health care facilities
Histology/FNAC	Simple, rapid, sensitive and invasive but well tolerated by most patients Can distinguish eumycetoma from actinomycetoma	Requires expert to perform the procedure A pathologist is required to interpret the results General or regional anaesthesia needed Biopsy procedure requires experts
Serology	Less invasive procedure Cheaper and less time consumed Useful for measuring therapeutic response	Cannot reliably diagnose mycetomca Pure antigens needed Cross reactivity is a common challenge
Molecular-PCR	Fast, reliable and easy identification of causative agents Important for studying epidemiology of mycetoma agents Useful for generating accurate therapeutic data	Expensive, not readily available in endemic areas Only available in tertiary facilities Inappropriate for use in the field
Molecular-LAMP	Reliable identification of causative agents Can be used in resource-limited settings Relatively cheaper and easier compared to PCR User friendly	Less specific than PCR in identifying etiologic agents, possibility of field application

*MRI* magnetic resonance imaging, *CT* computed tomography, *PCR* polymerase chain reaction, *FNAC* fine needle aspiration cytology, *LAMP* loop-mediated isothermal amplification

### Early phase of mycetoma

At early onset of the disease, a papule, a nodule, an abscess or just an induration lacking a clear margin can be seen. At this point, a meticulous history may reveal an episode of trauma or walking barefooted prior to presentation of symptom or sign. More often, this could be difficult to elicit since patients fail to notice such trivial events [47]. Though the precise incubation period of mycetoma is still unknown, the interval between initial infection to presentation in the health facility may vary from 3 months to 50 years [1]. The epidermis may become hypo- or hyper-pigmented and the nodules which increase in size eventually rupture along fascial plains to form secondary nodules [14]. These ruptured nodules continue as a sinus tract discharging fluid which contains grains, and is usually characterised by alternating episodes of healing and breakdown as the affected area progressively enlarges, becomes more oedematous, nodulated and deformed [47]. Pain is hardly felt at this stage and has only been reported in 15% of patients [8]. This absence of pain has been attributed to production of anaesthetic substances and only in rare cases, pain can results from super-imposed bacterial infection, bone expansion or nerve damage in the later part of the disease course [8].

### Late phase of mycetoma

Mycetoma patients usually present late with the classical characteristic triad of a painless subcutaneous mass, multiple sinus, and discharge containing grains [53]. This delay in presentation of patients to health centres in chronically deformed state is attributed to the painless, slowly progressive nature of the disease coupled with poor health education and low socio-economic status [54]. Vital structures such as tendons and nerves are usually well preserved until late in the disease course due to adequate supply of blood in mycetoma [2, 20]. Regional lymph nodes may enlarge as a result of superimposed bacterial infection or immune complex deposition [20]. Infection is usually confined except in patients with immuno-suppression or septicaemia [2]. Although diverse aetiological agents are implicated in mycetoma, the clinical presentations usually mimic one another, and apparently most cases may appear similar [1]. In spite of shared similarity, actinomycetoma tends to be more aggressive and destructive, rapidly proliferating, inflammatory and it invades bone faster than eumycetoma [14].

### Anatomical preference

As expected, mycetoma develops on body part which frequently comes into contact with the habitat of these saprophytic organisms. Foot is the most predominantly affected part of the body (82% of cases), followed by hand (7%) [20]. In cases where the foot is involved, the dorsum is frequently affected [2]. Other parts such as the knee, arm, leg, head, neck, thigh, perineum, chest, abdominal walls, facial bones, mandible, paranasal sinuses, eyelid, vulva, orbit, and scrotum are seldom affected [2]. Cysts may develop in situations where the elbow, knee and buttocks are involved [50]. In regions such as Mexico where labourers carry woods and other agricultural materials on their back, mycetoma commonly affects this area with subsequent complication of paraplegia due to direct spread to the vertebral bone and spinal cord [1]. Cases of mycetoma affecting the scalp [55–57] and cerebrum have also been documented [58–61].

### Mycetoma in pregnancy

The preponderance of mycetoma in male relative to female is a usual finding [15]. This discrepancy has stirred curiosity among researchers to unveil the factors responsible for this marked difference which has been explained as the physiological effects of progesterone levels in female that may have inhibitory effects on the proliferation of some causative agents [16]. Interestingly, contradictory to this thought, other researchers found mycetoma to be more active and aggressive during pregnancy [62, 63]. This aggression of mycetoma in gestation has been found to be associated with alteration in hormonal environment and suppressed immune response during pregnancy [2]. While some suggests hormonal effects, others attributed this sex variation to greater exposure (physical) of men than women to agricultural activities [1]. However, in some endemic regions, female are more committed to field work than men yet mycetoma is more prevalent in men supporting the idea of possible hormonal effect [2]. Therefore, the actual reasons behind this difference is still controversial.

### Mycetoma in children

Generally, mycetoma is thought to occur infrequently in children (reported incidence, 3.0–4.5%) and probably the youngest reported case was a 2-year-old boy from India [64]. Even though the clinical features, radiological, cytological and ultrasonography findings in children are not different from those seen in adults, the rate of amputations in children are however, lower which can be attributed to shorter duration of disease and early phase reporting to the health facility. In cases of amputation, children, however, are relatively more liable to become social outcasts, and are thus at increased risk of dropping out of school [1].

## Diagnosis

**Fig. 2:**
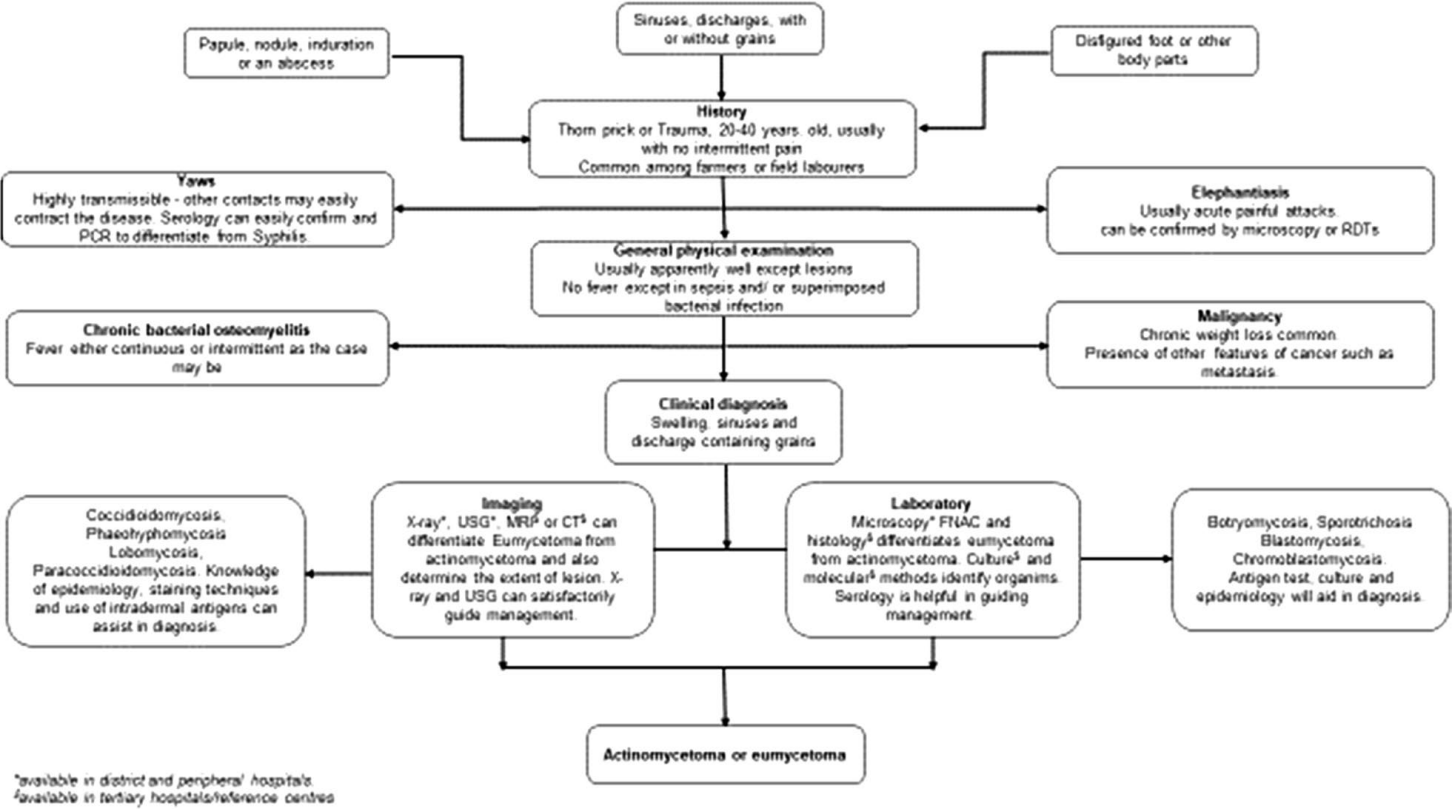
Diagnostic algorithm for mycetoma

A comprehensive summary on features of mycetoma is described and an algorithm which may guide a physician in approaching a suspected case of mycetoma is suggested (Fig. 2 and Table 2). Patients presenting with papule, nodule, induration, swelling or disfigured foot from endemic region need to be carefully scrutinised, and their age, sex and occupation ought to be kept in mind regarding its occurrence [65]. These patients rarely complain of pain unlike in elephantiasis where episodic acute painful attacks are experienced [66]. Yaws mimics mycetoma but due to its contagious nature, close contacts are likely to contract the disease easily and usually occur in childhood with 70% below the age 15 years [67]. Unlike mycetoma, the lesions are also painful and itchy in Yaws [67]. Where disease progression is rapid, accompanied by severe weight loss and systemic involvement, malignancy needs to be ruled out [68]. Chronic bacterial osteomyelitis may mimic it, but it is usually accompanied by fever and pain over affected sites unlike mycetoma except when superimposed by bacterial infection [69, 70]. Fungal diseases such as botryomycosis, sporotrichosis, blastomycosis, chromoblastomycosis, coccidioidomycosis, phaeohyphomycosis, lobomycosis and paracoccidioidomycosis have close resemblance to mycetoma [71]. Knowledge of their epidemiology and clinical progression will assist the physician in making a presumptive diagnosis. Focused investigation at various levels of facility also helps the physician to hit the diagnosis as shown in the algorithm (Fig. 2). Table 1 reveals findings and results in some of the available tests to confirm mycetoma and Table 3 summarizes the differences between actinomycetoma and eumycetoma. A disease condition producing chronically swollen and deformed foot characterised by draining sinuses should be considered as a differential diagnosis of mycetoma as shown in Table 4. Additionally, some important diagnostic tools, their pitfalls and the level of health care system to find them are provided in Tables 2 and 5 to aid in diagnosis.

**Table 3:** Major clinical differences between eumycetoma and actinomycetoma: summary of key findings

Characteristics	Eumycetoma	Actinomycetoma
Affected body parts [1]	Usually hand, feet and other parts of arms and legs	Usually chest, abdomen and head
Lesion [32]	Well encapsulated with a clear margin	Diffuse, no clear margin
Disease progression [64]	Slow	More rapid and inflammatory
Sinus morphology [1]	Proliferative, protuberant	Depressed, flat
Sinuses [32]	Few	Many
Bone invasion [32]	Delayed	Rapid
Bone radiograph [32]	Fewer but larger cavities with clear margins	Numerous, small cavities with unclear margins
Color and texture of grains	Different colors, but mostly white or black; coarse texture	Different colors but not black; fine texture
Lymphatic spread [1]	Occassional	Frequent
Drugs (e.g.) [49]	Antifungals (ketoconazole, voriconazole, posaconazole, etc.)	Antibiotics (sulfamethoxazole–trimethoprim, rifampicin, amikacin, etc.)
Recurrence [1]	More	Less

**Table 4:** Infectious and non-infectious diseases mimicking mycetoma: consideration for differential diagnosis

Infectious diseases	Non-infectious diseases
Parasitic	Tumours
Elephantiasis	Acral melanoma
Bacterial	Bone cyst
Actinomycosis	Fibro lipomas
Chronic bacterial osteomyelitis	Fibroma
Syphilis	Foreign body granuloma
Tuberculosis	Ganglion cyst
Yaws	Giant cell tumour
Fungal	Gouty typhus
Blastomycosis	Kaposi’s sarcoma
Botryomycosis	Lipomas
Chromoblastomycosis	Malignant melanoma
Coccidioidomycosis	Nerve sheath tumours
Lobomycosis	Osteosarcoma
Paracoccidioidomycosis	Rhabdomyosarcoma
Phaeohyphomycosis	Sarcomas (others)
Sporotrichosis	Subdermal abscess
	Occupational
	Podoconiosis

Disease names are given in alphabetical order

**Table 5:** Level of health care facilities and possible diagnostic approach for mycetoma

Facility	Method of diagnosis
Field	Clinical
Peripheral	Clinical, microscopy, radiology (X-ray)
District	Clinical, microscopy, radiology (X-ray, ultrasound)
Tertiary and referral centres	Clinical, microscopy, radiology (X-ray, ultrasound, MRI, CT), culture, histology, serology, molecular (PCR, LAMP)

*MRI* magnetic resonance imaging, *CT* computed tomography, *PCR* polymerase chain reaction, *LAMP* loop-mediated isothermal amplification

## Treatment

Treatment of mycetoma is still based on expert opinion in the absence of WHO treatment guideline.

Varying antimicrobial treatment options for actinomycetoma exist. Eumycetoma treatment usually poses a challenge. Antifungals are used, and in many cases, combination of surgery and antifungals are preferred [23]. Other treatment regimens and their outcomes are also shown in Additional file 1: Table S2.

## Actinomycetoma

Weilsh, modified Weilsh, two step regimens and other treatment modalities are available. In Weilsh regimen, 15 mg/kg IM of Amikacin in two divided doses + sulfamethoxazole (35 mg/kg/day) and trimethoprim (7 mg/kg/day) in three divided doses for 21 days at 1–3 cycles of 15-days intervals is used in the intensive phase. In its maintenance phase, trimethoprim and sulfamethoxazole (7 and 35 mg/kg/day, respectively) are administered for 2 weeks after the last cycle [18]. In modified Welsh, 15 mg/kg/day of Amikacin in divided doses + sulfamethoxazole–trimethoprim tablets (35 + 7 mg/kg/day) + rifampicin capsule (10 mg/kg/day) for 21 days at 1–3 cycles of 15 days intervals in the intensive phase is followed by sulfamethoxazole–trimethoprim tablets (35 + 7 mg/kg/day) + rifampicin capsule (10 mg/kg/day) for 3 months in the maintenance phase [49]. In two step modified regimen, Gentamicin (80 mg twice daily, IV), and cotrimoxazole (two tablets of 960 mg twice daily) for 4 weeks in the intensive phase is followed by Doxycycline (100 mg orally, twice daily) and cotrimoxazole (two tablets of 960 mg twice daily) which are given until 5–6 months after complete healing of all sinuses [49].

## Eumycetoma

Over 50% of cases treated with imidazoles and triazoles respond well to therapy, especially immunocompetent patients having infections limited to the subcutaneous tissues [23].

Treatment modalities include itraconazole (200–400 mg/day), ketoconazole (400 mg/day), voriconazole 400–600 mg/day, posaconazole (200 mg, four times daily), terbinafine (500–1000 mg/day), and amphotericin B (0.5–1.25 mg/kg/day) alone or with any combination(s) [49].

## Recommendation on treatment

Treatment of both eumycetoma and actinomycetoma is currently based on expert opinion. We encourage active research to establish treatment guideline for it.

## Prevention and control

**Fig. 3:**
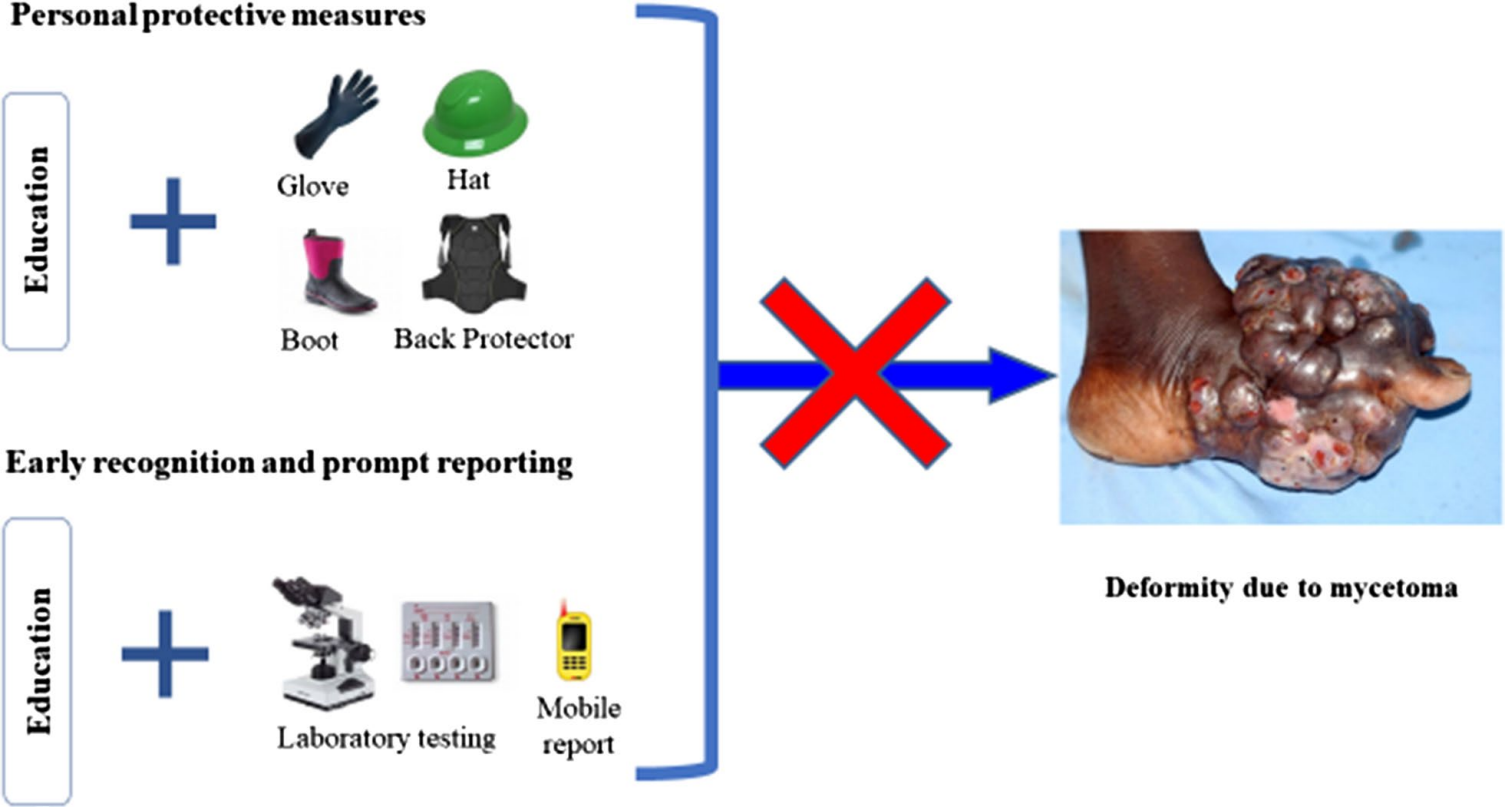
Recommended prevention and control strategies for mycetoma (Source of photograph of mycetoma affected leg: http://www.who.int/neglected_diseases/diseases/massive_foot_mycetoma.jpg)

Since there are no outlined prevention or control programs for mycetoma by WHO till date, we recommend aggressive formal and informal health education, and sensitization in endemic areas for community members regarding protection of exposed parts of the body (by using rubber boots and gloves) during work that are in contact with the habitat of offending organisms (Fig. 3 and Additional file 1). Early recognition and reporting of suspected cases will reduce severity of complication and improve treatment outcome.

## Conclusion

The late presentation of mycetoma patients results to gruesome complication. Early presentation, recognition, reporting and initiation of appropriate regimen is needful in reducing this disability. The clinical overview of mycetoma and the suggested algorithm may assist health workers to suspect and recognise mycetoma amidst other differentials in their community. Since no standard outlined treatment measure has been given by WHO, we have suggested covering of exposed parts of the body during working on the field as a preventive measure to avert contact with infective agents. The suggested preventive and control measures can be utilized by health workers/community leaders to educate people at risk to reduce their risk of exposure and also to report cases early. We recommend that Dermatological diseases with severe physical disability and social stigma leading to isolation like mycetoma needs to be given more attention in research and funding.

## Additional file

**Additional file 1.** Information on the causative agents of mycetoma (eumycetoma and actinomycetoma), and their current treatment algorithms.

## Abbreviations

WHO: World Health Organization
GMS: Grocotts Methenamine silver stain
B and B: Brown and Brenn stain
PAS: periodic-acid Schiff staining
MRI: magnetic resonance imaging
CT: computed tomography
PCR: polymerase chain reaction
LAMP: loop-mediated isothermal amplification
FNAC: fine needle aspiration cytology
IM: intramuscular
IV: intravenous

## References

[1] Zijlstra EE, van de Sande WW, Welsh O, el Mahgoub S, Goodfellow M, Fahal AH (2016). Mycetoma: a unique neglected tropical disease. Lancet Infect Dis..

[2] Fahal AH (2004). Mycetoma: a thorn in the flesh. Trans R Soc Trop Med Hyg..

[3] van’t Noordende AT, van Brakel WH, Banstola N, Dhakal KP (2016). The impact of leprosy on marital relationships and sexual health among married women in Eastern Nepal. J Trop Med..

[4] Zijlstra EE, van de Sande WW, Fahal AH (2016). Mycetoma: a long journey from neglect. PLoS Negl Trop Dis..

[5] Fahal A, el Mahgoub S, El Hassan AM, Abdel-Rahman ME, Alshambaty Y, Hashim A (2014). A new model for management of mycetoma in the Sudan. PLoS Negl Trop Dis..

[6] Gilquin JM, Riviere B, Jurado V, Audouy B, Kouatche JB, Bergeron E (2016). First case of actinomycetoma in france due to a novel nocardia species, *Nocardia boironii* sp. nov. mSphere..

[7] Mattioni S, Develoux M, Brun S, Martin A, Jaureguy F, Naggara N (2013). Management of mycetomas in France. Medecine et Maladies Infectieuses..

[8] Gosselink C, Thomas J, Brahmbhatt S, Patel NK, Vindas J (2008). Nocardiosis causing pedal actinomycetoma: a case report and review of the literature. J Foot Ankle Surg..

[9] Veraldi S, Grancini A, Venegoni L, Merlo V, Guanziroli E, Menicanit C (2016). Mycetoma caused by *Aspergillus nidulans*. Acta Dermato-Venereol..

[10] van de Sande WW (2013). Global burden of human mycetoma: a systematic review and meta-analysis. PLoS Negl Trop Dis..

[11] Mencarini J, Antonelli A, Scoccianti G, Bartolini L, Roselli G, Capanna R (2016). Madura foot in Europe: diagnosis of an autochthonous case by molecular approach and review of the literature. New Microbiol..

[12] Efared B, Tahiri L, Boubacar MS, Atsam-Ebang G, Hammas N, Hinde EF (2017). Mycetoma in a non-endemic area: a diagnostic challenge. BMC Clin Pathol..

[13] van de Sande WW, Fahal AH, Goodfellow M, el Mahgoub S, Welsh O, Zijlstra EE (2014). Merits and pitfalls of currently used diagnostic tools in mycetoma. PLoS Negl Trop Dis..

[14] Jimenez AL, Salvo NL (2011). Mycetoma or synovial sarcoma? A case report with review of the literature. J Foot Ankle Surg..

[15] Lupi O, Tyring SK, McGinnis MR (2005). Tropical dermatology: fungal tropical diseases. J Am Acad Dermatol..

[16] Lichon V, Khachemoune A (2006). Mycetoma: a review. Am J Clin Dermatol..

[17] Rattanavong S, Vongthongchit S, Bounphamala K, Vongphakdy P, Gubler J, Mayxay M (2012). Actinomycetoma in SE Asia: the first case from Laos and a review of the literature. BMC Infect Dis..

[18] Mathews S, Jadhav R, Reza A, Karim T (2012). Actinomycetoma-the welsh regimen in a rural Indian scenario. Indian J Surg..

[19] Welsh O, Al-Abdely HM, Salinas-Carmona MC, Fahal AH (2014). Mycetoma medical therapy. PLoS Negl Trop Dis..

[20] Fahal AH, Shaheen S, Jones DH (2014). The orthopaedic aspects of mycetoma. Bone Joint J..

[21] Ameen M (2009). Managing mycetomas. Trop Doc..

[22] Ameen M, Arenas R (2009). Developments in the management of mycetomas. Clin Exp Dermatol..

[23] Welsh O, Vera-Cabrera L, Salinas-Carmona MC (2007). Mycetoma. Clin Dermatol..

[24] Patel S, Sethi A (2009). Imported tropical diseases. Dermatol Ther..

[25] Maiti PK, Ray A, Bandyopadhyay S (2002). Epidemiological aspects of mycetoma from a retrospective study of 264 cases in West Bengal. Trop Med Int Health..

[26] Khatri ML, Al-Halali HM, Fouad Khalid M, Saif SA, Vyas MC (2002). Mycetoma in Yemen: clinicoepidemiologic and histopathologic study. Int J Dermatol..

[27] Bendl BJ, Mackey D, Al-Saati F, Sheth KV, Ofole SN, Bailey TM (1987). Mycetoma in Saudi Arabia. J Trop Med Hyg..

[28] Lopez Martinez R, Mendez Tovar LJ, Lavalle P, Welsh O, Saul A, Macotela Ruiz E (1992). Epidemiology of mycetoma in Mexico: study of 2105 cases. Gaceta Medica de Mexico..

[29] Perez-Blanco M, Hernandez-Valles R, Fernandez-Zeppenfeldt G, Yegres F (1996). Mycetoma: report of 3 cases in Falcon State, Venezuela. Invest Clin..

[30] Negroni R, Lopez Daneri G, Arechavala A, Bianchi MH, Robles AM (2006). Clinical and microbiological study of mycetomas at the Muniz hospital of Buenos Aires between 1989 and 2004. Revista Argentina de Microbiol..

[31] Castro LG, Belda Junior W, Salebian A, Cuce LC (1993). Mycetoma: a retrospective study of 41 cases seen in Sao Paulo, Brazil, from 1978 to 1989. Mycoses..

[32] Ahmed AO, van Leeuwen W, Fahal A, van de Sande W, Verbrugh H, van Belkum A (2004). Mycetoma caused by *Madurella mycetomatis*: a neglected infectious burden. Lancet Infect Dis..

[33] Destombes P, Mariat F, Rosati L, Segretain G (1977). Mycetoma in Somalia—results of a survey done from 1959 to 1964. Acta Tropica..

[34] Daoud M, Ezzine Sebai N, Badri T, Mokhtar I, Fazza B, Kamoun MR (2005). Mycetoma: retrospective study of 13 cases in Tunisia. Acta Dermatovenerologica Alpina, Pannonica, et Adriatica..

[35] Develoux M, Vetter JM, Audoin J, Treguer J (1985). 63 cases of mycetoma in the Niger Republic (etiological study based on histopathology). Bulletin de la Societe de Pathologie Exotique et de ses Filiales..

[36] Dieng MT, Niang SO, Diop B, Ndiaye B (2005). Actinomycetomas in Senegal: study of 90 cases. Bulletin de la Societe de Pathologie Exotique (1990)..

[37] Dieng MT, Sy MH, Diop BM, Niang SO, Ndiaye B (2003). Mycetoma: 130 cases. Ann Dermatol Venereol..

[38] Ndiaye B, Develoux M, Langlade MA, Kane A (1994). Actinomycotic mycetoma. Apropos of 27 cases in Dakar; medical treatment with cotrimoxazole. Ann Dermatol Venereol..

[39] Pelzer K, Tietz HJ, Sterry W, Haas N (2000). Isolation of both Sporothrix schenckii and Nocardia asteroides from a mycetoma of the forefoot. Br J Dermatol..

[40] Balabanoff VA (1980). Mycetomas originated from South-East Bulgaria (author’s transl). Annales de Parasitologie Humaine et Comparee..

[41] Rigopoulos D, Mavridou M, Nicolaidou E, Christofidou E, Antoniou C, Stratigos A (2000). Mycetoma due to actinomycetes: a rare entity in Europe. Int J Dermatol..

[42] Papaioannides D, Akritidis NK (2001). Painless foot swelling with a chronic purulent discharge. Western J Med..

[43] Ispoglou SS, Zormpala A, Androulaki A, Sipsas NV (2003). Madura foot due to Actinomadura madurae: imaging appearance. Clin Imaging..

[44] De Palma L, Marinelli M, Pavan M, Manso E, Ranaldi R (2006). A rare European case of madura foot due to actinomycetes. Joint Bone Spine.

[45] Buonfrate D, Gobbi F, Angheben A, Marocco S, Farina C, Van Den Ende J (2014). Autochthonous cases of mycetoma in Europe: report of two cases and review of literature. PLoS ONE..

[46] Gunduz K, Orguc S, Demireli P, Inanir I, Surucuoglu S, Ovali GY (2006). A case of mycetoma successfully treated with itraconazole and co-trimoxazole. Mycoses..

[47] Green WO, Adams TE (1964). Mycetoma in the United States; a review and report of seven additional cases. Am J Clin Pathol..

[48] Polden KE, Jehle H (2017). Actinomycosis of the foot—a South African case. S Afr J Surg Suid-Afrikaanse tydskrif vir chirurgie..

[49] Verma P, Jha A (2018). Mycetoma: reviewing a neglected disease. Clinical and experimental dermatology..

[50] Zarei Mahmoudabadi A, Zarrin M (2008). Mycetomas in Iran: a review article. Mycopathologia..

[51] Mahgoub ES, Gumaa SA, El Hassan AM (1977). Immunological status of mycetoma patients. Bull de la Societe de Pathologie Exotique et de ses Filiales..

[52] Verwer PE, Notenboom CC, Eadie K, Fahal AH, Verbrugh HA, van de Sande WW (2015). A Polymorphism in the chitotriosidase gene associated with risk of mycetoma due to *Madurella mycetomatis* mycetoma—a retrospective study. PLoS Negl Trop Dis..

[53] Suleiman SH, el Wadaella S, Fahal AH (2016). The surgical treatment of mycetoma. PLoS Negl Trop Dis..

[54] van de Sande WW, el Maghoub S, Fahal AH, Goodfellow M, Welsh O, Zijlstra E (2014). The mycetoma knowledge gap: identification of research priorities. PLoS Negl Trop Dis..

[55] Welsh O, Morales-Toquero A, Vera-Cabrera L, Vazquez-Martinez O, Gomez-Flores M, Ocampo-Candiani J (2011). Actinomycetoma of the scalp after a car accident. Int J Dermatol..

[56] Shanbhag NU, Karandikar S, Deshmukkh PA (2010). Disseminated orbital actinomycetoma: a case report. Indian J Ophthalmol..

[57] Patil SP, Gautam MM, Sodha AA, Khan KJ (2009). Primary cutaneous nocardiosis with craniocerebral extension: a case report. Dermatol Online J..

[58] Natarajan M, Balakrishnan D, Muthu AK, Arumugham K (1975). Maduromycosis of the brain. Case report. J Neurosurg..

[59] Sundaram C, Umabala P, Laxmi V, Purohit AK, Prasad VS, Panigrahi M (2006). Pathology of fungal infections of the central nervous system: 17 years’ experience from Southern India. Histopathology..

[60] Beeram V, Challa S, Vannemreddy P (2008). Cerebral mycetoma with cranial osteomyelitis. J Neurosurg Pediatr..

[61] Maheshwari S, Figueiredo A, Narurkar S, Goel A (2010). Madurella mycetoma—a rare case with cranial extension. World Neurosurg..

[62] Sampaio FM, Galhardo MC, De FariasCardoso R, de OliveiraCoelho JM, Lyra MR, de Valle AC (2015). Eumycetoma on the foot caused by *Madurella mycetomatis*: amputation after significant worsening during pregnancy. Acta Dermato-Venereol..

[63] Sampaio FM, Wanke B, Freitas DF, Coelho JM, Galhardo MC, Lyra MR (2017). Review of 21 cases of mycetoma from 1991 to 2014 in Rio de Janeiro, Brazil. PLoS Negl Trop Dis..

[64] Joshi A, Acharya S, Anehosur VS, Tayaar AS, Gopalkrishnan K (2014). Oral eumycetoma of infancy: a rare presentation and a brief review. J Cranio-Maxillo-Facial Surg..

[65] Nazzaro G, Veraldi S (2018). Mycetomas: the experience of the dermatology unit of the University of Milan. Dermatopathology (Basel, Switzerland).

[66] Akogun OB, Akogun MK, Apake E, Kale OO (2011). Rapid community identification, pain and distress associated with lymphoedema and adenolymphangitis due to lymphatic filariasis in resource-limited communities of North-eastern Nigeria. Acta Tropica..

[67] Boock AU, Awah PK, Mou F, Nichter M (2017). Yaws resurgence in Bankim, Cameroon: the relative effectiveness of different means of detection in rural communities. PLoS Negl Trop Dis..

[68] Mattox TW (2017). Cancer cachexia: cause, diagnosis, and treatment. Nutr Clin Pract..

[69] Calhoun JH, Manring MM (2005). Adult osteomyelitis. Infect Dis Clin North Am..

[70] Stone B, Street M, Leigh W, Crawford H (2016). Pediatric tibial osteomyelitis. J Pediatr Orthop..

[71] Rivitti EA, Aoki V (1999). Deep fungal infections in tropical countries. Clin Dermatol..

