# Supplementary material for: Mycetoma: a clinical dilemma in resource limited settings
Source: Ann Clin Microbiol Antimicrob. 2018 Aug 10;17:35. doi: 10.1186/s12941-018-0287-4 (PMC6085652; doi:10.1186/s12941-018-0287-4)
Supplement: Supplementary file 1 — Additional file 1. Information on the causative agents of mycetoma (eumycetoma and actinomycetoma), and their current treatment algorithms. [file 12941_2018_287_MOESM1_ESM.docx]

**Additional file 1:** **Information on the causative agents of mycetoma (eumycetoma and actinomycetoma), and their current treatment algorithms**

**Mycetoma — a clinical dilemma in resource limited settings**

Pembi Emmanuel^1,2,3^, Shyam Prakash Dumre^1^, Stephen John^4^, Juntra Karbwang^5*^, Kenji Hirayama^1,6^

^1^Department of Immunogenetics, Institute of Tropical Medicine (NEKKEN), Nagasaki University, Nagasaki, Japan

^2^Program for Nurturing Global leaders in Tropical and Emerging communicable Diseases, Graduate school of Biomedical sciences, Nagasaki University.

^3^Hospital Services Management Board Yola, Adamawa State Ministry of Health, Nigeria

^4^Adamawa State Agency for HIV/AIDS Control, Yola, Nigeria

^5^Department of Clinical Product Development, Institute of Tropical Medicine (NEKKEN), Nagasaki University, Nagasaki, Japan

^6^Neglected Tropical Diseases Innovation (NTDi) Center, Institute of Tropical Medicine (NEKKEN), Nagasaki University, Nagasaki, Japan

**Author e-mails:**

Pembi Emmanuel (PE) - pembiemmanuel@gmail.com

Shyam Prakash Dumre(SPD) - sp.dumre@gmail.com

Stephen John(SJ) - wizemannstv2@gmail.com

Kenji Hirayama(KH) - hiraken@nagasaki-u.ac.jp

karbwangj@nagasaki-u.ac.jp (JKL) - karbwangj@nagasaki-u.ac.jp

**Correspondence to:**

Juntra Karbwang, M.D., Ph.D

Head, Department of Clinical Product Development

Institute of Tropical Medicine (NEKKEN), Nagasaki University, 1-12-4 Sakamoto

Nagasaki 852-8523, Japan

(E-mail: karbwangj@nagasaki-u.ac.jp)

**Table 1. Causative agents of mycetoma**

| **Eumycetoma** | **Actinomycetoma** |
| --- | --- |
| *Aspergillus ﬂavus*^1^ | *Nocardia asteroids*^2^ |
| *Aspergillus nidulans*^3^ | *Nocarida brasiliensis*^2^ |
| *Acremonium kiliense*^4^ | *Nocardia farcinica^5^* |
| Acremonium recifei^4^ | *Nocardia nova*^6^ |
| *Cladosporium carrionii*^7^ | *Nocardia otitidiscaviarum* (*Nocardia* caviae)^8^ |
| *Curvularia lunata^9^* | *Nocardia transvalensis*^10^ |
| *Cylindrocarpon spp*^11^ | *Nocardia yamanashiensis*^12^ |
| *Diaporthe phaseolorum (Phomopsis phaseoli)*^13^ | *Nocardia takedensis*^14^ |
| *Exophiala jeanselmei^15^* | *Nocardia veterana*^16^ |
| *Falciformispora (Leptosphaeria) senegalensis*^17^ | *Actinomyces israeli^5^* |
| *Falciformispora (Leptosphaeria) thompkinsii*^17^ | *Actinomadura pelletierii^18^* |
| *Fusarium solani*^19^ | *Actinomadura madurae^18^* |
| *Fusarium oxysoprum^20^* | *Streptomyces somaliensis^20^* |
| *Fusarium falciforme (Acremonium falciforme)*^21^ |  |
| *Fusarium sublutinans*^22^ |  |
| *Madurella mycetomatis*^23,24^ |  |
| *Trematosphaeria grisea (Madurella grisea)*^25,26^ |  |
| *Madurella pseudomycetomatis*^9^ |  |
| *Medicopsis romeroi (Pyrenochaeta romeroi)^27^* |  |
| *Microsporum audouinii*^28^ |  |
| *Neotestudina rosatii^4^* |  |
| *Neoscytalidium dimidiatum (Scytalidium dimidiatumm)*^1,15^ |  |
| *Paecilomyces lilacinus*^29^ |  |
| *Phialophora jeanselmei*^29^ |  |
| *Pseudochaetosphaeronema larense^20^* |  |
| *Scedosporium boydii (Pseudallescheria and Petriellidium boydii)*^30^ |  |
| *Scedosporium apiospermum*^31-33^ |  |

List of references are given at the end of the document.

**Table 2. Eumycetoma treatment: present scenario**

| **Drug** | **Dose** | **Cure** | **Country** |
| --- | --- | --- | --- |
| Ketoconazale | 200-400 mg once daily for 1 year + surgery | 40-70% | Sudan and India^34,35^ |
| Terbinafine | Terbinafine (500 mg twice daily) for 24-48 weeks | 25% | Senegal^36^ |
| Posaconaconazole | Posaconaconazole (800 mg once daily) for 24 months | 80% | Argentina^37^ |

List of references are given at the end of the document.

**Table 3. Actinomycetoma treatment: current scenario**

| **Drug** | **dose** | **cure** | **country** |
| --- | --- | --- | --- |
| Oral TMP/SMX | (800 mg/160 mg) once daily for a minimum of 1 | 60-90% | Sudan, Mexico and Senegal^38-40^. |
| Parenteral streptomycin+  Oral TMP/SMX | Streptomycin (14 mg/kg once daily) +  TMP/SMX (800 mg/160) mg once daily | 63% | Sudan^41^ |
| Parenteral gentamicin | 80 mg twice daily (for weeks then doxycycline (200 mg once daily) and TMP/SMX (800 mg/160 mg twice daily) given throughout therapy | 66% | India^42^ |
| Parenteral amikacin+  Oral TMP/SMX | Parenteral amikacin (15mg/kg once daily for 3 weeks cycle, up to 4 weeks) plus oral TMP/SMX (800 mg/160 mg once daily) was administered | 95% | Mexico^43^ |
| Parenteral imipenem,  amikacin,  Oral TMP/SMX | Parenteral imipenem (1.5g once daily) plus amikacin (15mg/kg once daily) for 3 weeks cycle repeated every 6 months and oral TMP/SMX (800 mg/160 mg once daily) | 40% | mexco^44^ |
| Linezolid | 600mg twice daily for 2 months | Case report | USA^45^ |
| Rifampicin+ TMP/SMX | Rifampicin (600 mg once daily) and TMP/SMX (800 mg/160 mg twice daily) for 10 months | Case report | India^46^, |
| Amoxicillin/clavulanic acid | dose of 875/125mg twice daily | 71% | Mexico^47^ |

SMX, sulfamethoxazole; TMP,trimethoprim

List of references are given at the end of the document.

**References**

1. Padhi S, Uppin SG, Uppin MS, et al. Mycetoma in South India: retrospective analysis of 13 cases and description of two cases caused by unusual pathogens: Neoscytalidium dimidiatum and Aspergillus flavus. International journal of dermatology 2010;49:1289-96.

2. Soto-Mendoza N, Bonifaz A. Head actinomycetoma with a double aetiology, caused by Nocardia brasiliensis and N. asteroides. The British journal of dermatology 2000;143:192-4.

3. van de Sande WW. Global burden of human mycetoma: a systematic review and meta-analysis. PLoS neglected tropical diseases 2013;7:e2550.

4. Hay RJ, Mackenzie DW. The histopathological features of pale grain eumycetoma. Transactions of the Royal Society of Tropical Medicine and Hygiene 1982;76:839-44.

5. Ahmed AO, van Leeuwen W, Fahal A, van de Sande W, Verbrugh H, van Belkum A. Mycetoma caused by Madurella mycetomatis: a neglected infectious burden. The Lancet Infectious diseases 2004;4:566-74.

6. Arora G, Friedman M, Macdermott RP. Disseminated Nocardia nova infection. Southern medical journal 2010;103:1269-71.

7. Mahaisavariya P, Chaiprasert A, Sivayathorn A, Khemngern S. Deep fungal and higher bacterial skin infections in Thailand: clinical manifestations and treatment regimens. International journal of dermatology 1999;38:279-84.

8. Shahapur PR, Peerapur BV, Shahapur RP, Honnutagi RM, Biradar MS. Lymphocutaneous nocardiosis caused by Nocardia otitidiscaviarum: A case report and review of literature. Journal of natural science, biology, and medicine 2014;5:197-201.

9. Yan J, Deng J, Zhou CJ, Zhong BY, Hao F. Phenotypic and molecular characterization of Madurella pseudomycetomatis sp. nov., a novel opportunistic fungus possibly causing black-grain mycetoma. Journal of clinical microbiology 2010;48:251-7.

10. Mirza SH, Campbell C. Mycetoma caused by Nocardia transvalensis. Journal of clinical pathology 1994;47:85-6.

11. Hemashettar BM, Siddaramappa B, Padhye AA, Sigler L, Chandler FW. White grain mycetoma caused by a Cylindrocarpon sp. in India. Journal of clinical microbiology 2000;38:4288-91.

12. Mitja O, Hays R, Van Straten C, Robson J, Koka M, Bassat Q. Mycetoma caused by Nocardia yamanashiensis, Papua New Guinea. The American journal of tropical medicine and hygiene 2012;86:1043-5.

13. Iriart X, Binois R, Fior A, et al. Eumycetoma caused by Diaporthe phaseolorum (Phomopsis phaseoli): a case report and a mini-review of Diaporthe/Phomopsis spp invasive infections in humans. Clinical microbiology and infection : the official publication of the European Society of Clinical Microbiology and Infectious Diseases 2011;17:1492-4.

14. Kresch-Tronik NS, Carrillo-Casas EM, Arenas R, et al. First case of mycetoma associated with Nocardia takedensis. The Journal of dermatology 2013;40:135-6.

15. Aamir S, Aman S, Haroon T. Mycetoma caused by Scytalidium dimidiatum. The British journal of dermatology 2003;148:174-6.

16. Kashima M, Kano R, Mikami Y, et al. A successfully treated case of mycetoma due to Nocardia veterana. The British journal of dermatology 2005;152:1349-52.

17. Machmachi H, Godineau N, Develoux M, et al. Black grain mycetoma caused by Leptosphaeria tompkinsii. Medical mycology 2011;49:186-9.

18. Welsh O, Morales-Toquero A, Vera-Cabrera L, Vazquez-Martinez O, Gomez-Flores M, Ocampo-Candiani J. Actinomycetoma of the scalp after a car accident. International journal of dermatology 2011;50:854-7.

19. Yera H, Bougnoux ME, Jeanrot C, Baixench MT, De Pinieux G, Dupouy-Camet J. Mycetoma of the foot caused by Fusarium solani: identification of the etiologic agent by DNA sequencing. Journal of clinical microbiology 2003;41:1805-8.

20. Welsh O, Vera-Cabrera L, Salinas-Carmona MC. Mycetoma. Clinics in dermatology 2007;25:195-202.

21. Xiujiao X, Hong S, Ai-e X. Eumycetoma due to Acremonium falciforme acquired in China. Mycoses 2012;55:e4-7.

22. Campos-Macias P, Arenas-Guzman R, Hernandez-Hernandez F. Fusarium subglutinans: A new eumycetoma agent. Medical mycology case reports 2013;2:128-31.

23. Verdolini R, Amerio P, Bugatti L, et al. Madura's foot: report of a case caused by Madurella mycetomatis. European journal of dermatology : EJD 2000;10:627-9.

24. Fahal AH, Rahman IA, El-Hassan AM, Rahman ME, Zijlstra EE. The safety and efficacy of itraconazole for the treatment of patients with eumycetoma due to Madurella mycetomatis. Transactions of the Royal Society of Tropical Medicine and Hygiene 2011;105:127-32.

25. Montes LF, Freeman RG, McClarin W. Maduromycosis due to Madurella grisea. Report of the fifth North American case. Archives of dermatology 1969;99:74-9.

26. Severo LC, Vetoratto G, Oliveira Fde M, Londero AT. Eumycetoma by Madurella grisea. Report of the first case observed in the southern Brazilian region. Revista do Instituto de Medicina Tropical de Sao Paulo 1999;41:139-42.

27. Ahmed SA, van den Ende BH, Fahal AH, van de Sande WW, de Hoog GS. Rapid identification of black grain eumycetoma causative agents using rolling circle amplification. PLoS neglected tropical diseases 2014;8:e3368.

28. West BC, Kwon-Chung KJ. Mycetoma caused by microsporum audouinii. First reported case. American journal of clinical pathology 1980;73:447-54.

29. Motswaledi HM, Mathekga K, Sein PP, Nemutavhanani DL. Paecilomyces lilacinus eumycetoma. International journal of dermatology 2009;48:858-61.

30. Horre R, Schumacher G, Marklein G, et al. Mycetoma due to Pseudallescheria boydii and co-isolation of Nocardia abscessus in a patient injured in road accident. Medical mycology 2002;40:525-7.

31. Gupta MK, Banerjee T, Kumar D, Rastogi A, Tilak R. White grain mycetoma caused by Scedosporium apiospermum in North India: a case report. The international journal of lower extremity wounds 2013;12:286-8.

32. Boyce Z, Collins N. Scedosporium apiospermum: An unreported cause of fungal sporotrichoid-like lymphocutaneous infection in Australia and review of the literature. The Australasian journal of dermatology 2015;56:e39-42.

33. Oliveira Fde M, Unis G, Hochhegger B, Severo LC. Scedosporium apiospermum eumycetoma successfully treated with oral voriconazole: report of a case and review of the Brazilian reports on scedosporiosis. Revista do Instituto de Medicina Tropical de Sao Paulo 2013;55:121-3.

34. Mahgoub ES, Gumaa SA. Ketoconazole in the treatment of eumycetoma due to Madurella mycetomii. Transactions of the Royal Society of Tropical Medicine and Hygiene 1984;78:376-9.

35. Venugopal PV, Venugopal TV. Treatment of eumycetoma with ketoconazole. The Australasian journal of dermatology 1993;34:27-9.

36. N'Diaye B, Dieng MT, Perez A, Stockmeyer M, Bakshi R. Clinical efficacy and safety of oral terbinafine in fungal mycetoma. International journal of dermatology 2006;45:154-7.

37. Negroni R, Tobon A, Bustamante B, Shikanai-Yasuda MA, Patino H, Restrepo A. Posaconazole treatment of refractory eumycetoma and chromoblastomycosis. Revista do Instituto de Medicina Tropical de Sao Paulo 2005;47:339-46.

38. Welsh O, Salinas MC, Rodriguez MA. Treatment of eumycetoma and actinomycetoma. Current topics in medical mycology 1995;6:47-71.

39. Mahgoub ES. Treatment of actinomycetoma with sulphamethoxazole plus trimethoprim. The American journal of tropical medicine and hygiene 1972;21:332-5.

40. Dieng MT, Niang SO, Diop B, Ndiaye B. [Actinomycetomas in Senegal: study of 90 cases]. Bulletin de la Societe de pathologie exotique (1990) 2005;98:18-20.

41. Mahgoub ES. Medical management of mycetoma. Bulletin of the World Health Organization 1976;54:303-10.

42. Ramam M, Bhat R, Garg T, et al. A modified two-step treatment for actinomycetoma. Indian journal of dermatology, venereology and leprology 2007;73:235-9.

43. Welsh O, Sauceda E, Gonzalez J, Ocampo J. Amikacin alone and in combination with trimethoprim-sulfamethoxazole in the treatment of actinomycotic mycetoma. Journal of the American Academy of Dermatology 1987;17:443-8.

44. Fuentes A, Arenas R, Reyes M, Fernandez RF, Zacarias R. [Actinomycetoma and Nocardia sp. Report of five cases treated with imipenem or imipenem plus amikacin]. Gaceta medica de Mexico 2006;142:247-52.

45. Moylett EH, Pacheco SE, Brown-Elliott BA, et al. Clinical experience with linezolid for the treatment of nocardia infection. Clinical infectious diseases : an official publication of the Infectious Diseases Society of America 2003;36:313-8.

46. Joshi R. Treatment of actinomycetoma with combination of rifampicin and co-trimoxazole. Indian journal of dermatology, venereology and leprology 2008;74:166-8; author reply 8.

47. Bonifaz A, Flores P, Saul A, Carrasco-Gerard E, Ponce RM. Treatment of actinomycetoma due to Nocardia spp. with amoxicillin-clavulanate. The British journal of dermatology 2007;156:308-11.
